# Anemia and long-term outcomes after second-generation drug-eluting stent implantation: a retrospective cohort study of mortality and clinical restenosis

**DOI:** 10.1186/s43044-026-00725-8

**Published:** 2026-04-28

**Authors:** Mani Ghorbanzadeh-Aghdam, Samaneh Sardari, Mahsa Nikkhoo, Seyedhesamoddin Khatami, Amirali Zarrin, Mobina Riahi, Mojtaba Salarifar

**Affiliations:** 1 Tehran Heart Center, Tehran University of Medical Sciences, Tehran, Islamic Republic of Iran; 2 Skin Research Center, Shahid Beheshti University of Medical Sciences, Tehran, Islamic Republic of Iran; 3 Physiology Research Center, Iran University of Medical Sciences, Tehran, Islamic Republic of Iran; 4 Faculty of Medicine, Tehran University of Medical Sciences, Tehran, Islamic Republic of Iran

**Keywords:** Anemia, Percutaneous coronary intervention, Drug-eluting stents, Target lesion revascularization, In-stent restenosis, Mortality

## Abstract

**Background:**

Anemia is common in patients undergoing percutaneous coronary intervention (PCI) and is a well-established predictor of bleeding and mortality. However, whether anemia contributes to *clinically relevant* in-stent restenosis (ISR) in the era of second-generation drug-eluting stents (DES) and competing long-term risks remains uncertain.

**Methods:**

We retrospectively analyzed 4,117 consecutive adults treated with PCI using second-generation DES at Tehran Heart Center. Anemia was defined as hemoglobin <13 g/dL in men and < 12 g/dL in women. The prespecified primary endpoint was clinically driven target lesion revascularization (TLR) within 5 years, used as a pragmatic proxy for clinical ISR and requiring ischemic symptoms or objective ischemia attributable to the index lesion. Secondary endpoints included all-cause mortality and major adverse cardiac and cerebrovascular events (MACCE). Associations between anemia and outcomes were estimated using Cox proportional hazards models with inverse probability weighting (IPW) based on baseline covariates; Fine–Gray competing-risk models treating death as a competing event were prespecified sensitivity analyses for TLR.

**Results:**

Anemia was present in 369/4,117 (9.0%) patients. Cumulative TLR occurred in 73 (1.9%) patients, with only 3 events in the anemic group. Given this low event rate, statistical power to detect a difference in TLR was limited. TLR rates did not differ significantly between groups (IPW-adjusted HR 0.27, 95% CI 0.07 to 1.01; *p* = 0.051), a finding that was consistent in competing-risk analyses. In contrast, mortality was significantly higher in anemic patients (11.5% vs. 4.6%; IPW-adjusted HR 1.87, 95% CI 1.22 to 2.87; *p* = 0.004).

**Conclusions:**

In an all-comers cohort undergoing PCI with second-generation DES, baseline anemia was not associated with an increased risk of clinically driven TLR, but it remained a strong independent predictor of long-term mortality. These findings suggest that in contemporary practice, the dominant prognostic impact of anemia is on mortality. However, given the low TLR event rate, the relationship between anemia and clinically manifest restenosis requires further investigation in larger, adequately powered studies.

**Supplementary Information:**

The online version contains supplementary material available at 10.1186/s43044-026-00725-8.

## Introduction

Coronary artery disease (CAD) remains the leading cause of morbidity and mortality worldwide, accounting for more than 16% of all deaths globally [1] and over one-quarter of annual deaths in Iran [2]. Primary percutaneous coronary intervention (PPCI) is the established treatment for ST-elevation myocardial infarction and has significantly improved survival outcomes. Despite these advances, adverse long-term events continue to challenge clinicians [3].

In-stent restenosis (ISR), defined as recurrent luminal narrowing in a previously stented coronary segment, typically occurs within the first year after PCI [4]. Although the incidence of ISR has declined with the advent of newer-generation drug-eluting stents (DES), it still affects 5–10% of patients and can result in recurrent angina, repeat revascularization, or myocardial infarction [5]. Predictors of ISR include patient-related comorbidities, lesion complexity, and procedural characteristics, yet outcomes remain heterogeneous across populations [5, 6].

Anemia, reported in 10–30% of patients undergoing PCI [7], has been associated with increased risks of bleeding, major adverse cardiac and cerebrovascular events (MACCE), and mortality [8]. Mechanistically, reduced oxygen-carrying capacity, heightened systemic inflammation, and impaired endothelial repair may contribute to poor outcomes [9]. Several studies have also suggested that anemia predisposes to ISR (7–9). Hussein et al. (8) demonstrated a four-fold higher incidence of clinical ISR among anemic patients, while Hu et al. [7] reported anemia as an independent predictor of angiographic ISR. However, other investigations, particularly in the DES era, have not confirmed this association, highlighting ongoing uncertainty about the role of anemia in ISR [10, 11].

Despite multiple reports linking anemia to ISR in the bare-metal and first-generation DES eras, contemporary data in all-comer populations treated exclusively with second-generation DES and clinically driven ISR endpoints remain scarce and inconsistent. Moreover, few studies have explicitly contrasted anemia’s impact on local restenosis with its well-established association with long-term mortality under competing long-term risks. Therefore, we aimed to determine whether baseline anemia independently predicts clinically driven ISR, as measured by target lesion revascularization (TLR), and long-term all-cause mortality in a large, unselected cohort of patients undergoing PCI with second-generation DES. We hypothesized that anemia would remain a powerful systemic prognostic marker for mortality but would not independently increase clinically relevant ISR.

## Methods

### Study design and population

**Fig. 1:**
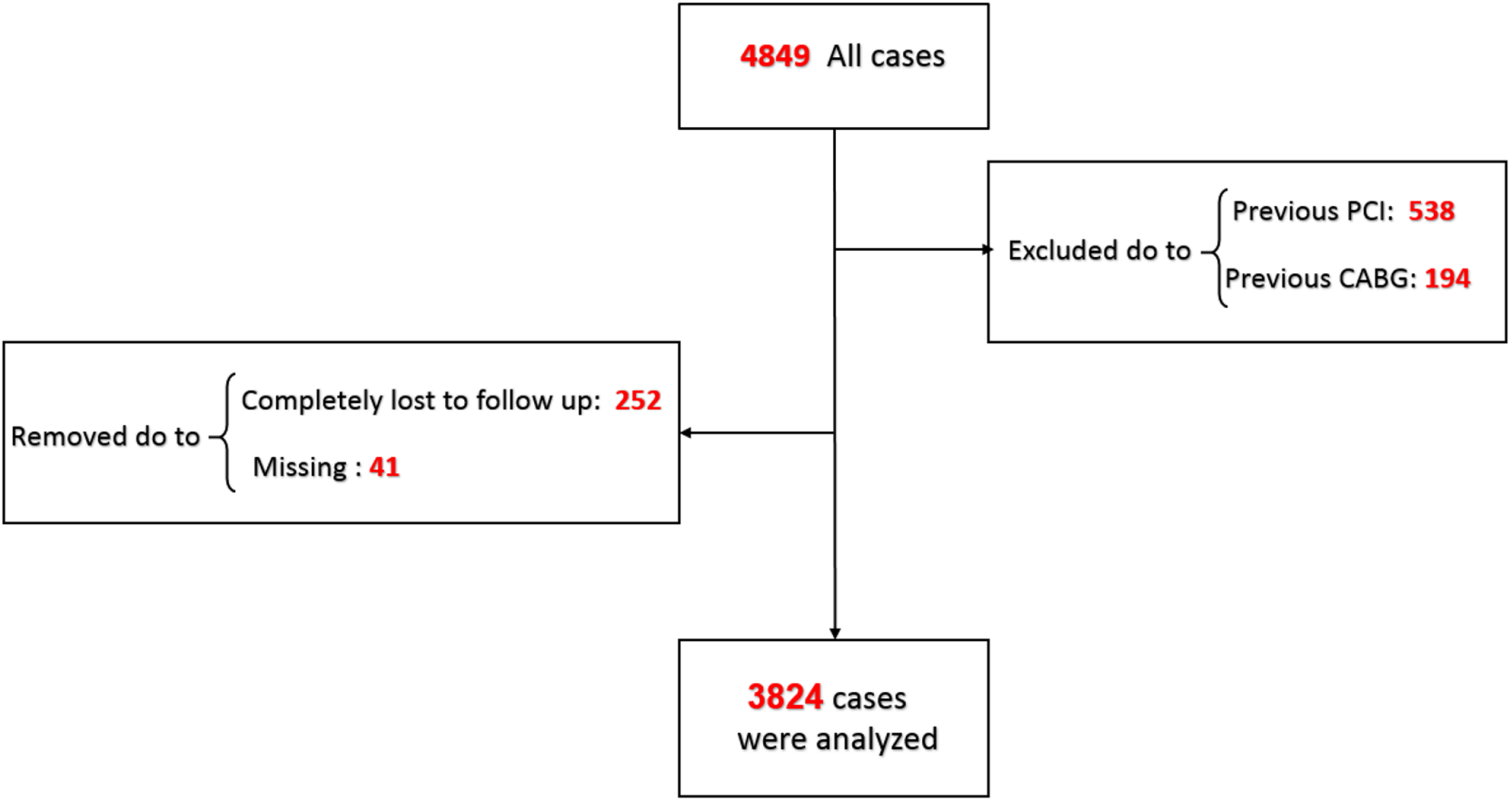
Study flowchart of patient selection. Flow diagram showing patient inclusion and exclusion. Of 4,849 screened cases, exclusions were made for prior PCI, prior CABG, loss to follow-up, and missing data, resulting in 4,117 patients included in the study cohort (3,748 without anemia and 369 with anemia); time-to-event analyses were restricted to patients with available follow-up and complete covariate data (*N* = 3,824)

We performed a retrospective, single-center cohort study at Tehran Heart Center including consecutive adults (≥ 18 years) who underwent percutaneous coronary intervention (PCI) with second-generation drug-eluting stents (DES). Both primary PCI for ST-elevation myocardial infarction and non-primary PCI (urgent/elective) were eligible. We excluded patients with chronic total occlusion, left main disease, end-stage renal disease, decompensated liver disease, active malignancy, prior PCI or prior CABG before the index procedure, and those with missing follow-up or key baseline data (Fig. 1), to focus on a de-novo lesion cohort and reduce heterogeneity related to advanced comorbidity and lesion subsets; history of myocardial infarction was permitted provided no prior PCI or CABG had been performed. This observational cohort study was reported in accordance with the STROBE statement; the completed checklist is provided in Supplementary File 1.

### Definitions

Anemia was defined as hemoglobin < 13 g/dL in men and < 12 g/dL in women. Chronic kidney disease (CKD) was defined as an estimated glomerular filtration rate (eGFR) < 60 mL/min/1.73 m² or a documented history of renal failure. The prespecified primary endpoint was clinically driven target lesion revascularization (TLR) within 5 years, used as a pragmatic proxy for clinical in-stent restenosis (ISR). Clinically driven TLR required ischemic symptoms or objective evidence of ischemia attributable to the index lesion and angiographic confirmation of a significant stenosis in the previously stented segment. At our center, follow-up coronary angiography is not performed routinely after PCI and is instead symptom-driven or guided by objective evidence of ischemia; however, the registry did not systematically capture the overall proportion of patients undergoing follow-up angiography or the indication categories, and this could not be quantified. Consequently, clinically driven TLR captures only restenosis events that become clinically manifest and require repeat revascularization. Objective evidence of ischemia was defined as ischemic changes on stress electrocardiography, stress-induced wall motion abnormalities on echocardiography, or a positive finding on myocardial perfusion imaging or invasive physiologic assessment (e.g., fractional flow reserve ≤ 0.80). Routine surveillance angiography was not performed.

Secondary endpoints were all-cause mortality, major adverse cardiac and cerebrovascular events (MACCE), and target vessel revascularization (TVR). Given the known heterogeneity in composite cardiovascular endpoint definitions across observational studies, MACCE was explicitly prespecified in the present study as all-cause death, myocardial infarction, unstable angina, stroke, or any repeat revascularization [13].

### Data collection

Data were extracted from the Tehran Heart registry using a structured checklist. Collected variables included demographic characteristics, cardiovascular risk factors (diabetes mellitus, hypertension, dyslipidemia, smoking, family history of CAD), procedural details (stent length, diameter, number of stents, lesion location, bifurcation involvement, post-dilation, and TIMI flow before and after PCI), and clinical outcomes. Missing information was obtained through telephone follow-up when necessary.

### Follow-up and outcomes

Patients were followed for up to five years after the index procedure. The primary outcome was the incidence of clinically driven ISR, operationalized as TLR meeting the above clinical and angiographic criteria. Secondary outcomes included all-cause mortality, MACCE, and TVR. Follow-up data were obtained from medical records, outpatient visits, and structured telephone interviews. Of note, the center does not perform routine angiographic follow-up; angiography was performed only when clinically indicated by symptoms or objective evidence of ischemia. Mortality data were obtained from medical records, direct contact with families during telephone follow-up, and linkage with national death registries where available.

### Statistical analysis

Continuous variables were expressed as mean ± standard deviation (SD) or median with interquartile range (IQR) and compared using the independent t-test or Wilcoxon rank-sum test, as appropriate. Categorical variables were reported as counts and percentages and compared using the chi-square and/or Fisher’s exact test, as appropriate.

A post-hoc power calculation was performed for the primary endpoint of TLR. With the observed cumulative incidence of 1.9% and 73 events (including only 3 events in the anemic group), the study had limited statistical power to detect clinically meaningful between-group differences; a minimum detectable effect size was not estimated, and no simulation-based power analysis was performed.

Cox proportional hazards regression models were used to evaluate the effect of anemia on TLR and mortality. To minimize confounding, inverse probability weighting (IPW) based on baseline covariates was applied. The IPW model included 9 baseline covariate terms (age, sex, BMI, diabetes, hypertension, dyslipidemia, smoking, cardiovascular history, and history of renal failure) to limit model complexity given 73 TLR events (approximately 8 events per covariate term); a minimum detectable effect size was not estimated, and no simulation-based power analysis was performed. Although eGFR is presented in Table 1 for descriptive purposes, it was not incorporated into the IPW model due to the limited number of TLR events (*n* = 73) to avoid model overfitting. History of renal failure was considered a clinically relevant proxy for significant kidney dysfunction.

For the primary endpoint of TLR, we prespecified Fine-Gray competing risk models, with all-cause death treated as a competing event. The results of these models are presented as subdistribution hazard ratios (SHR) with 95% confidence intervals (see Supplementary Table 1); formal assessments of proportionality and goodness-of-fit were not performed. Kaplan-Meier curves were generated for TLR-free and overall survival and compared using the log-rank test.

A two-sided *p* < 0.05 was considered statistically significant; no adjustment for multiple comparisons was planned, and secondary endpoints were interpreted as exploratory. For each endpoint, we analyzed time to first occurrence. Accordingly, the count of TLR as first MACCE (*n* = 36) differs from the cumulative TLR events over follow-up (*n* = 73); time-to-event analyses were restricted to patients with available follow-up. Analyses and Kaplan-Meier estimates are based on time-to-first-event. Analyses were performed using SPSS version 24 (IBM Corp., Armonk, NY, USA).

**Table 1:** Baseline characteristics of patients according to anemia status

Anemia					
Characteristic	Overall, *N* = 4,117	No, *N* = 3,748	Yes, *N* = 369	p-value	
Gender: *Female*	901 (21.9%)	770 (20.5%)	131 (35.5%)	**< 0.001**	
Diabetes	1,707 (41.5%)	1,516 (40.4%)	191 (51.8%)	**< 0.001**	
Dyslipidemia	2,168 (52.7%)	1,990 (53.1%)	178 (48.2%)	0.075	
Hypertension	1,770 (43.0%)	1,573 (42.0%)	197 (53.4%)	**< 0.001**	
Current smoking	1,581 (38.4%)	1,481 (39.5%)	100 (27.1%)	**< 0.001**	
Opium overuse	234 (6.4%)	218 (6.5%)	16 (4.9%)	0.260	
*Missing*	450	406	44		
Positive family history of CAD	787 (19.1%)	725 (19.3%)	62 (16.8%)	0.245	
*Missing*	1	0	1		
Previous MI (without prior PCI/CABG)	327 (7.9%)	296 (7.9%)	31 (8.4%)	0.733	
History CVA /TIA	125 (3.0%)	108 (2.9%)	17 (4.6%)	0.066	
*Missing*	6	6	0		
History renal failure	55 (1.3%)	32 (0.9%)	23 (6.2%)	**< 0.001**	
eGFR (mL/min/1.73 m²)	82.4 ± 16.7	83.9 ± 15.8	67.2 ± 19.4	**< 0.001**	
*Missing*	52	44	8		
History chronic lung disease	61 (1.5%)	50 (1.3%)	11 (3.0%)	**0.013**	
*Missing*	10	10	0		
History CHF	85 (2.1%)	71 (1.9%)	14 (3.8%)	**0.015**	
*Missing*	10	10	0		
Reduced LVEF (< 40%)	1,011 (27.3%)	899 (26.7%)	112 (33.5%)	**0.007**	
*Missing*	411	376	35		
Age	58.85 ± 11.70	58.29 ± 11.54	64.54 ± 11.76	**< 0.001**	
EF				**< 0.001**	
*Median (IQR)*	42.5 (35.0, 47.5)	42.5 (35.0, 47.5)	40.0 (35.0, 45.0)		
*Missing*	411	376	35		
BMI	28.00 ± 4.58	28.09 ± 4.56	27.09 ± 4.72	**< 0.001**	
*Missing*	11	10	1		
Primary PCI for STEMI	2,972 (72.2%)	2,704 (72.1%)	268 (72.6%)	0.843	
LAD territory	2,254 (54.7%)	2,081 (55.5%)	173 (46.9%)	**0.001**	
ACC/AHA lesion class				0.059	
*A or B1*	302 (7.3%)	284 (7.6%)	18 (4.9%)		
*B2 or C*	3,811 (92.7%)	3,461 (92.4%)	350 (95.1%)		
*Missing*	4	3	1		
Thrombus suction	340 (8.3%)	302 (8.1%)	38 (10.3%)	0.136	
Bifurcation lesion (vessel 1)	653 (15.9%)	603 (16.1%)	50 (13.6%)	0.203	
Lesion length (vessel 1), mm				**0.044**	
*Median (IQR)*	26.0 (20.0, 36.0)	26.0 (19.0, 36.0)	28.0 (21.0, 38.0)		
*Missing*	16	15	1		
First stent length				**0.025**	
*Median (IQR)*	28.0 (22.0, 37.0)	28.0 (20.0, 37.0)	29.0 (23.0, 38.0)		
*Missing*	10	9	1		
No of stents				0.197	
1	4,050 (98.4%)	3,690 (98.5%)	360 (97.6%)		
> 1	67 (1.6%)	58 (1.5%)	9 (2.4%)		
Stent diameter				0.067	
*Median (IQR)*	3.0 (2.8, 3.5)	3.0 (2.8, 3.5)	3.0 (2.5, 3.5)		
*Missing*	138	127	11		
Overlap Stent	238 (5.8%)	212 (5.7%)	26 (7.0%)	0.275	
Post dilation	2,708 (65.8%)	2,478 (66.1%)	230 (62.3%)	0.144	
Simultaneous PCI	704 (17.1%)	629 (16.8%)	75 (20.3%)	0.085	

Baseline demographic, clinical, and procedural characteristics of patients undergoing PCI with drug-eluting stents, stratified by presence of anemia. Continuous variables are presented as mean ± standard deviation (SD) or median (interquartile range), and categorical variables as number (percentage)

*p*-values were derived from chi-square or Fisher’s exact test for categorical variables and independent *t*-test or Wilcoxon rank-sum test for continuous variables, as appropriate

ACC/AHA lesion class was adjudicated by the operator at the time of PCI

### Ethical considerations

The study was conducted in accordance with the principles of the Declaration of Helsinki and was approved by the Research Ethics Committee of Tehran University of Medical Sciences. All patient data were fully anonymized prior to analysis to ensure confidentiality. Given the retrospective design and use of de-identified registry data, the requirement for individual informed consent was waived by the committee in accordance with institutional and national regulations.

## Results

### Baseline characteristics

A total of 4,117 patients were included, of whom 369 (9.0%) had anemia. Baseline characteristics stratified by anemia status are summarized in Table 1. Patients with anemia were older (64.5 ± 11.8 vs. 58.3 ± 11.5 years; *p* < 0.001), more frequently female (35.5% vs. 20.5%; *p* < 0.001), and had a higher prevalence of diabetes mellitus (51.8% vs. 40.4%; *p* < 0.001) and hypertension (53.4% vs. 42.0%; *p* < 0.001). Current smoking was less common among anemic patients (27.1% vs. 39.5%; *p* < 0.001). They also had higher rates of chronic kidney disease (6.2% vs. 0.9%; *p* < 0.001) and reduced ejection fraction (33.5% vs. 26.7%; *p* = 0.007). The median left ventricular ejection fraction was lower in the anemia group (40.0% [35.0–45.0] vs. 42.5% [35.0–47.5]; *p* < 0.001). Other comorbidities and procedural details are provided in Table 1. Overall, anemic patients presented with greater comorbidity burden and lower ejection fraction.

### Clinical outcomes

Clinical follow-up data are presented in Table 2. As the center does not perform routine angiographic follow-up, the reported TLR rate reflects only events that led to a clinical visit and subsequent angiography. Consequently, the overall incidence of clinically driven TLR (used here as a proxy for clinically manifest ISR) in our cohort was low (1.9%), and asymptomatic or minimally symptomatic angiographic ISR may have been missed. The overall incidence of first MACCE was 17.7%. Mortality as the first MACCE was more frequent in anemic patients (9.5% vs. 4.0%). Over a median follow-up of five years, cumulative TLR occurred in 73 patients (1.9%); when restricted to first MACCE, TLR was the initial event in 36 patients (0.9%). The proportion with TLR did not differ between anemic and non-anemic patients (cumulative TLR 0.9% vs. 2.0%, respectively). Mortality was higher among anemic patients (11.5% vs. 4.6%). In-stent events such as restenosis (0.1%) and thrombosis (0.2%) were rare and occurred only in the non-anemic group; timing categories (acute, subacute, late, and very late) were not captured and therefore were not reported. Importantly, the absolute incidence of clinically driven TLR was low in both groups, reflecting contemporary practice with second-generation DES and the focus on symptom-driven rather than routine angiographic surveillance.

**Table 2:** Clinical outcomes within 5 years by anemia status (First MACCE vs. cumulative events)

Anemia				
Characteristic	Overall, *N* = 4,117	No, *N* = 3,748	Yes, *N* = 369	
First MACCE				
* No*	3,180 (82.3%)	2,908 (82.7%)	272 (78.2%)	
* MI*	144 (3.7%)	126 (3.6%)	18 (5.2%)	
* UA*	166 (4.3%)	152 (4.3%)	14 (4.0%)	
* TLR*	36 (0.9%)	35 (1.0%)	1 (0.3%)	
*TVR*	16 (0.4%)	16 (0.5%)	0 (0.0%)	
* PCI*	114 (2.9%)	106 (3.0%)	8 (2.3%)	
* CABG*	32 (0.8%)	30 (0.9%)	2 (0.6%)	
* Mortality*	175 (4.5%)	142 (4.0%)	33 (9.5%)	
* CVA*	2 (0.1%)	2 (0.1%)	0 (0.0%)	
* Missing*	252	231	21	
Survival				
* Alive*	3,663 (94.8%)	3,355 (95.4%)	308 (88.5%)	
* Deceased*	202 (5.2%)	162 (4.6%)	40 (11.5%)	
* Missing*	252	231	21	
TLR	73 (1.9%)	70 (2.0%)	3 (0.9%)	
* Missing*	252	231	21	
In-stent thrombosis / restenosis (vessel 1)*				
* No*	4,106 (99.7%)	3,737 (99.7%)	369 (100.0%)	
*Restenosis*	3 (0.1%)	3 (0.1%)	0 (0.0%)	
* Thrombosis*	8 (0.2%)	8 (0.2%)	0 (0.0%)	

Clinical outcomes during five-year follow-up in patients undergoing PCI with drug-eluting stents, stratified by anemia status. Outcomes include first major adverse cardiac and cerebrovascular events (MACCE), individual event components (myocardial infarction, unstable angina, target lesion revascularization [TLR], target vessel revascularization [TVR], PCI, CABG, mortality, stroke), and overall survival. Data are expressed as number (percentage). “First MACCE” reflects the first occurring event per patient; cumulative rows reflect all events observed during follow-up

* Restenosis in this row denotes angiographic restenosis recorded outside the TLR definition; events without ischemia were not counted toward the clinically driven TLR endpoint (clinical ISR proxy)

### Univariate cox regression

Results of univariate Cox proportional hazards regression are shown in Table 3. Anemia was not significantly associated with TLR (HR 0.45, 95% CI 0.14–1.44; *p* = 0.180). In contrast, anemia was strongly associated with increased mortality (HR 2.53, 95% CI 1.78–3.58; *p* < 0.001). Other predictors of mortality included older age, female sex, diabetes, hypertension, chronic kidney disease, and reduced ejection fraction.

**Table 3:** Univariate cox regression analysis for target lesion revascularization and all-cause mortality

Characteristic	TLR		Univariate Cox regression			Overall survival		Univariate Cox regression		
	No, *N* = 3,753	Yes, *N* = 71	HR	95% CI	*p*-value	Alive, *N* = 3,623	Deceased, *N* = 201	HR	95% CI	*p*-value
Anemia	341 (9.1%)	3 (4.2%)	0.45	0.14, 1.44	0.180	304 (8.4%)	40 (19.9%)	2.53	1.78, 3.58	**< 0.001**
Age	58.79 ± 11.67	58.06 ± 11.31	1.00	0.98, 1.02	0.776	58.33 ± 11.45	66.79 ± 12.64	1.06	1.05, 1.08	**< 0.001**
BMI	28.02 ± 4.51	28.27 ± 3.97	1.00	0.96, 1.06	0.861	28.05 ± 4.49	27.50 ± 4.66	0.97	0.94, 1.00	**0.034**
Gender: *Female*	831 (22.1%)	15 (21.1%)	0.92	0.52, 1.62	0.768	785 (21.7%)	61 (30.3%)	1.50	1.11, 2.02	**0.009**
Diabetes	1,564 (41.7%)	26 (36.6%)	0.82	0.50, 1.32	0.412	1,477 (40.8%)	113 (56.2%)	1.78	1.35, 2.36	**< 0.001**
Dyslipidemia	1,990 (53.0%)	36 (50.7%)	0.95	0.60, 1.51	0.828	1,935 (53.4%)	91 (45.3%)	0.74	0.56, 0.98	**0.033**
Hypertension	1,606 (42.8%)	36 (50.7%)	1.41	0.88, 2.24	0.150	1,542 (42.6%)	100 (49.8%)	1.35	1.02, 1.78	**0.034**
Current smoking	1,437 (38.3%)	26 (36.6%)	0.87	0.54, 1.41	0.569	1,391 (38.4%)	72 (35.8%)	0.85	0.63, 1.13	0.254
Positive FH of CAD	717 (19.1%)	8 (11.3%)	0.58	0.28, 1.22	0.153	704 (19.4%)	21 (10.4%)	0.55	0.35, 0.86	**0.009**
Previous MI (without prior PCI/CABG)	287 (7.6%)	15 (21.1%)	3.00	1.69, 5.31	**< 0.001**	281 (7.8%)	21 (10.4%)	1.38	0.88, 2.17	0.161
History CVA/TIA	116 (3.1%)	3 (4.2%)	1.47	0.46, 4.67	0.518	108 (3.0%)	11 (5.5%)	1.78	0.97, 3.28	0.063
History Renal failure	49 (1.3%)	2 (2.8%)	2.24	0.55, 9.13	0.262	37 (1.0%)	14 (7.0%)	5.70	3.31, 9.82	**< 0.001**
History chronic lung disease	56 (1.5%)	0 (0.0%)	0.00	0.00, Inf	0.994	50 (1.4%)	6 (3.0%)	2.52	1.12, 5.68	**0.026**
History CHF	71 (1.9%)	1 (1.4%)	0.78	0.11, 5.66	0.810	53 (1.5%)	19 (9.5%)	5.89	3.67, 9.45	**< 0.001**
LAD territory	2,044 (54.5%)	39 (54.9%)	1.10	0.69, 1.75	0.702	1,973 (54.5%)	110 (54.7%)	1.06	0.80, 1.40	0.698
ACC/AHA lesion class										
*A or B1*	276 (7.4%)	3 (4.2%)				266 (7.3%)	13 (6.5%)			
*B2 or C*	3,477 (92.6%)	68 (95.8%)	1.69	0.53, 5.38	0.374	3,357 (92.7%)	188 (93.5%)	1.07	0.61, 1.87	0.826
Thrombus suction	290 (7.7%)	6 (8.5%)	1.17	0.51, 2.71	0.708	274 (7.6%)	22 (10.9%)	1.59	1.02, 2.49	**0.042**
Bifurcation lesion (vessel 1)	589 (15.7%)	12 (16.9%)	1.06	0.57, 1.97	0.859	561 (15.5%)	40 (19.9%)	1.26	0.89, 1.78	0.192
Lesion length (vessel 1), mm			1.01	0.99, 1.03	0.233			1.01	1.00, 1.02	**0.032**
*Median (IQR)*	26.0 (20.0, 36.0)	26.0 (20.5, 36.0)				26.0 (20.0, 36.0)	30.0 (21.0, 36.0)			
Overlap Stent	218 (5.8%)	8 (11.3%)	1.89	0.90, 3.95	0.091	209 (5.8%)	17 (8.5%)	1.34	0.82, 2.21	0.244
Post dilation	2,475 (65.9%)	44 (62.0%)	0.89	0.55, 1.44	0.643	2,396 (66.1%)	123 (61.2%)	0.83	0.62, 1.10	0.191

Univariate Cox proportional hazards regression evaluating the association of baseline anemia and other clinical variables with target lesion revascularization (TLR) and All-cause mortality over five years. Data are presented as hazard ratio (HR) with 95% confidence interval (CI) and corresponding *p*-values

### IPW-adjusted analysis

**Fig. 2:**
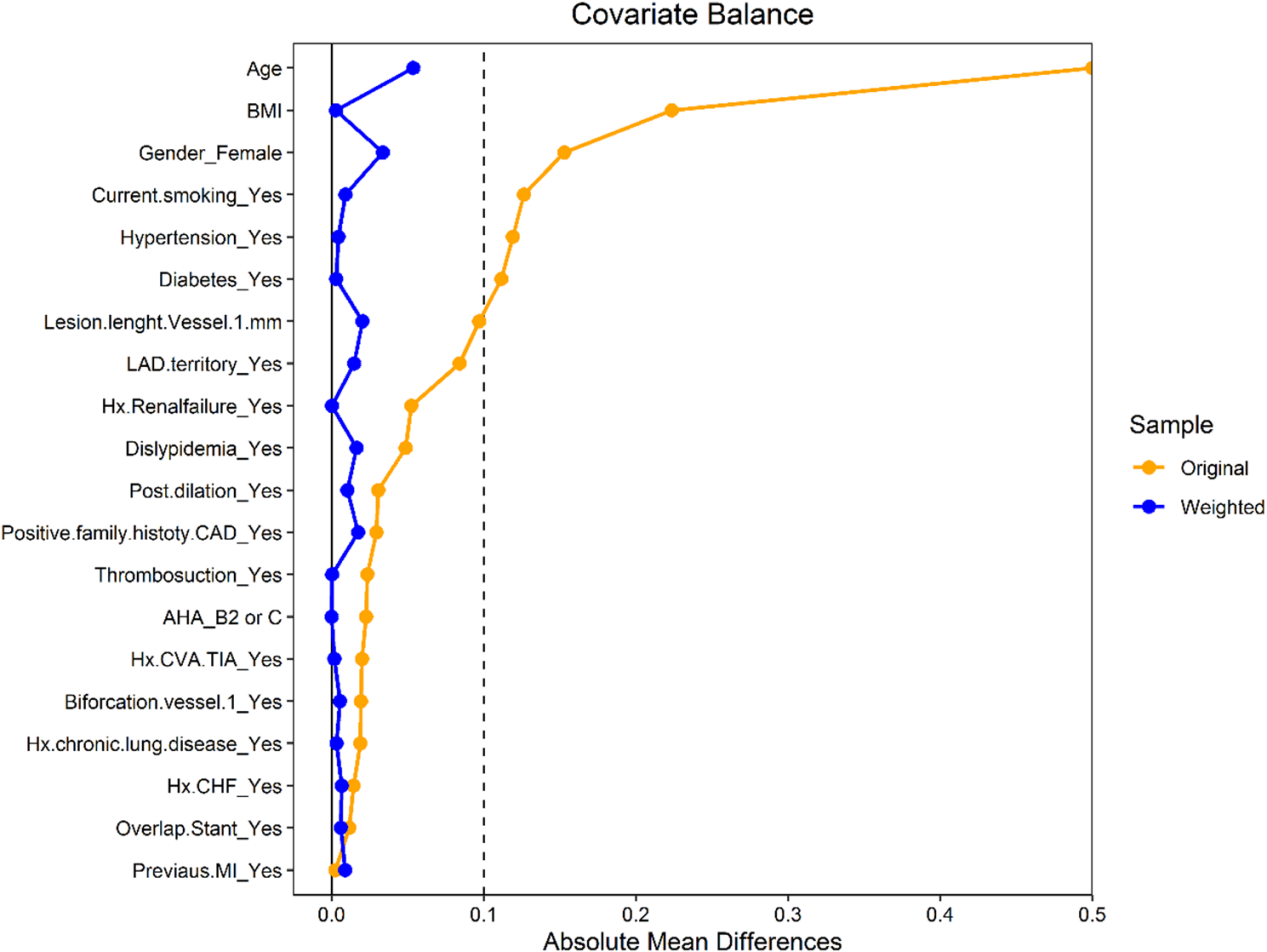
Covariate balance assessment before and after inverse probability weighting. Absolute standardized mean differences (SMD) of baseline covariates between patients with and without anemia before (Original) and after weighting (Weighted). After weighting, all covariate differences were < 10% (dashed line), indicating adequate balance. After IPW with stabilized and truncated weights, all SMDs were < 0.10, indicating adequate balance

After applying inverse probability weighting (IPW) to balance baseline covariates, the association between anemia and TLR remained non-significant (HR 0.27, 95% CI 0.07–1.01; *p* = 0.051). However, anemia remained an independent predictor of all-cause mortality (HR 1.87, 95% CI 1.22–2.87; *p* = 0.004). These findings are summarized in Table 4. In Fine–Gray competing-risk models treating death as a competing event, anemia was not associated with the subdistribution hazard of TLR (SHR 0.38, 95% CI 0.11–1.31; *p* = 0.124), indicating that the primary finding for clinically driven TLR was unchanged when accounting for competing mortality (see Supplementary Table 1). Covariate balance assessment by standardized mean differences before and after weighting is displayed in Fig. 2, demonstrating adequate covariate balance (< 10%).

**Table 4:** Effect of anemia on TLR and All-cause mortality: cox models before/after IPW

Outcome	Before weighting			After weighting		
	HR^*^	95% CI^*^	*p*-value	HR^*^	95% CI^*^	*p*-value
TLR	0.45	0.14, 1.44	0.180	0.27	0.07, 1.01	0.051
All-Cause Mortality	2.53	1.78, 3.58	**< 0.001**	1.87	1.22, 2.87	**0.004**

Hazard ratios (HR) with 95% confidence intervals (CI) and *p*-values for the association between baseline anemia and outcomes. Analyses are presented both before and after application of inverse probability weighting (IPW) to adjust for baseline covariates

### Kaplan–Meier survival curves

**Fig. 3:**
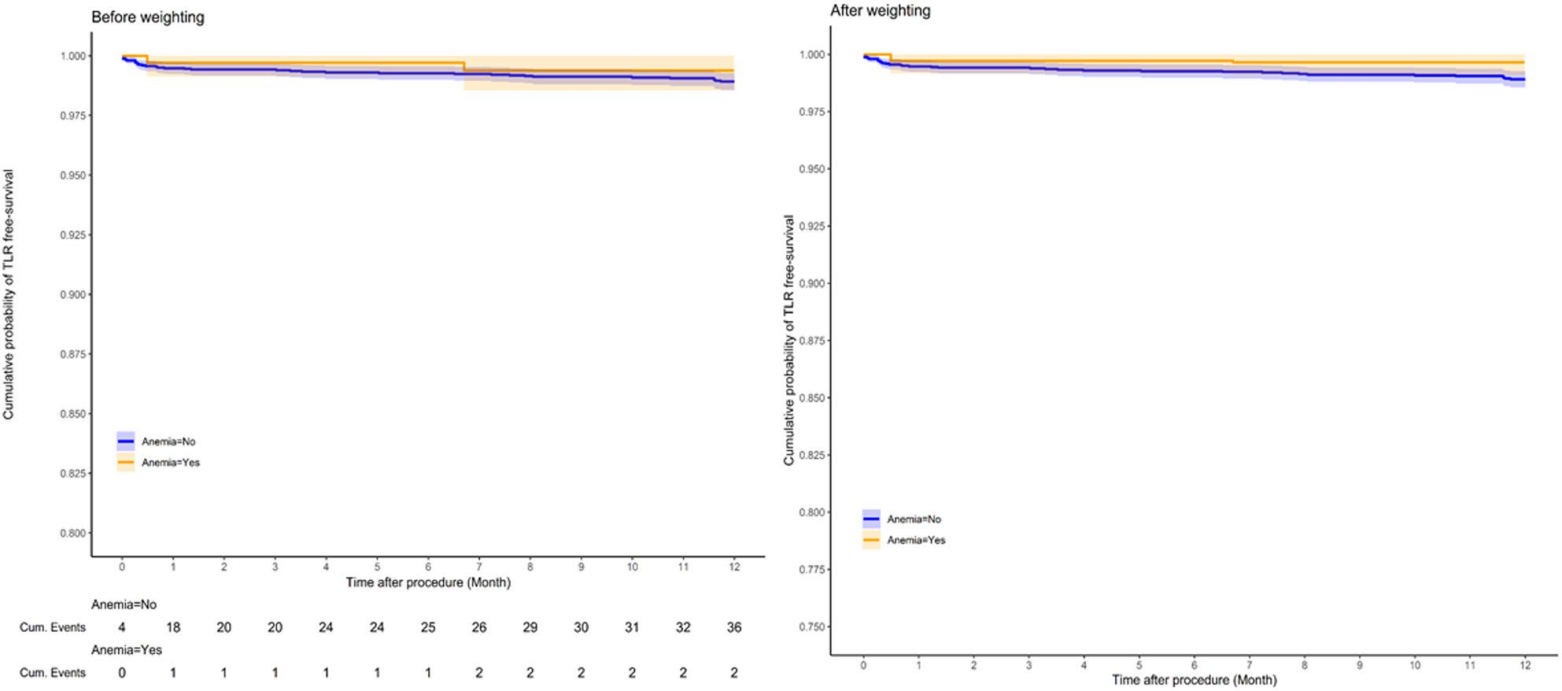
Kaplan–Meier Curves for Target Lesion Revascularization (TLR). Weighted Kaplan–Meier curves for TLR-free survival by anemia status before and after IPW; no between-group difference (log-rank *p* > 0.05). Numbers at risk are shown below each panel

**Fig. 4:**
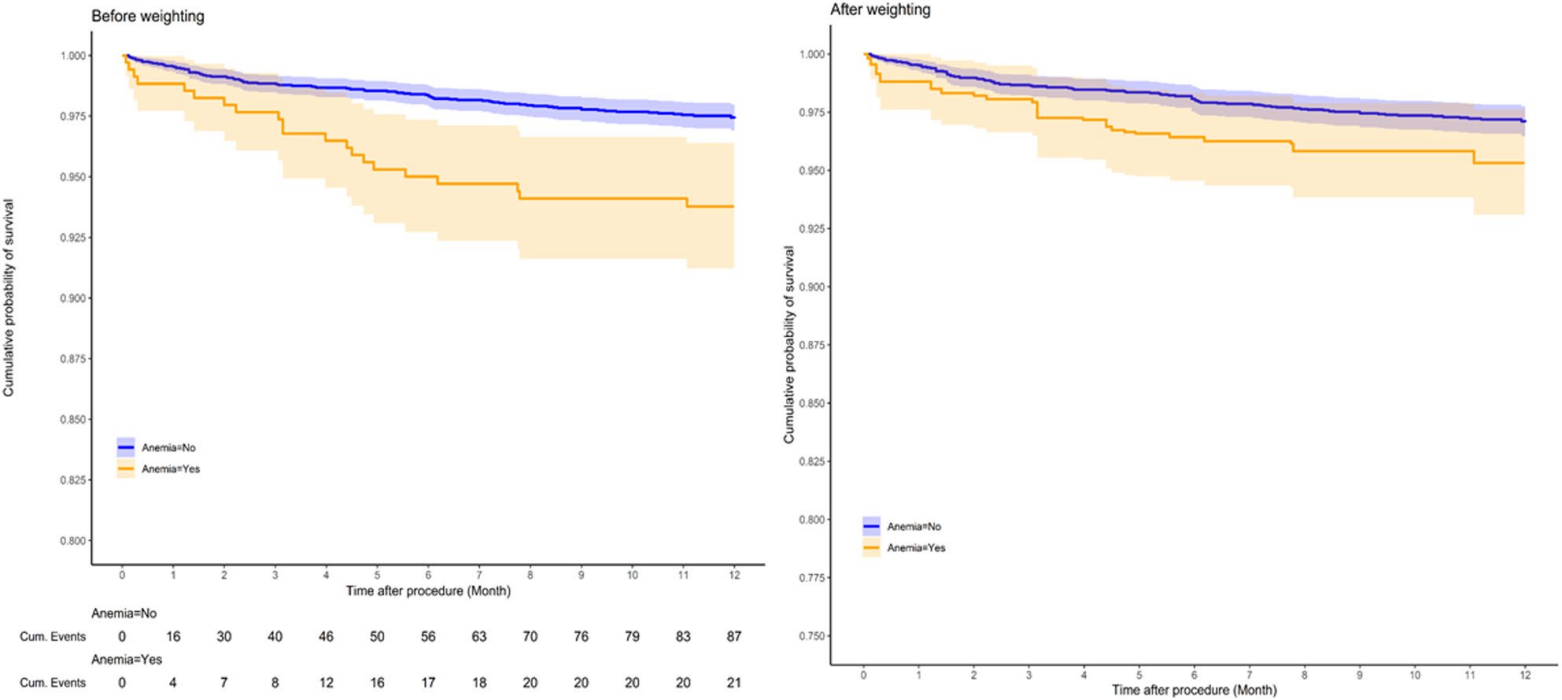
Kaplan–Meier Curves for Overall Survival. Weighted Kaplan–Meier curves for overall survival by anemia status before and after IPW; survival was lower with anemia (log-rank *p* < 0.001 before weighting; *p* = 0.004 after weighting). Numbers at risk are shown below each panel

Kaplan–Meier analyses are presented in Figs. 3 and 4. There was no significant difference in TLR-free survival between patients with and without anemia (Fig. 3; *p* > 0.05). In contrast, overall survival was significantly lower in the anemia group (Fig. 4; log-rank *p* < 0.001 before weighting; *p* = 0.004 after weighting). The number of patients at risk at each interval is displayed beneath each curve.

## Discussion

In this large, contemporary cohort of patients undergoing PCI with second-generation DES, baseline anemia was independently associated with a nearly two-fold higher risk of long-term all-cause mortality. However, despite rigorous adjustment, we did not find a statistically significant association between anemia and the incidence of clinically driven TLR over five years of follow-up. This null finding for our primary endpoint must be interpreted with caution due to the low overall event rate (*n* = 73, 1.9%), which resulted in limited statistical power, and the potential for under-ascertainment of asymptomatic restenosis in the absence of routine angiographic surveillance. Given the low absolute number of TLR events, the study was statistically underpowered to exclude modest or moderate associations between anemia and clinically driven TLR. Therefore, the absence of statistical significance should not be interpreted as definitive evidence of no association. The absence of an association persisted after IPW and in competing-risk analyses, suggesting that within these constraints, anemia does not appear to be a major driver of clinically manifest stent failure requiring repeat intervention. The very low rate of clinically driven TLR in our cohort (1.9%) is consistent with the excellent efficacy of second-generation DES but also highlights a key limitation of our primary endpoint definition. By relying on symptom-driven rather than routine angiographic follow-up, we inevitably underestimated the true burden of angiographic ISR. This is particularly relevant when comparing our results to older studies that used routine angiographic follow-up and reported higher ISR rates. Furthermore, with only 73 TLR events, and just 3 in the anemic group, our study was statistically underpowered to detect a modest but potentially clinically meaningful difference between groups. The wide confidence interval (0.07–1.01) and borderline p-value (0.051) for the adjusted HR reflect this instability and preclude a definitive conclusion that no association exists.

Our results contrast with several earlier studies that reported anemia as a significant predictor of ISR. Hussein et al. [8] observed a four-fold increase in clinical ISR among anemic patients undergoing PCI in the Egyptian Heart Journal cohort, and Hu et al. [7] found anemia to be an independent predictor of angiographic ISR in a Chinese population. Similarly, De Luca et al. [14] and Lee et al. [15] suggested that low hemoglobin was associated with a higher risk of restenosis in earlier stent eras. These reports raised the possibility that anemia might directly influence vascular healing and neointimal proliferation, positioning anemia as a potential local risk factor for restenosis.

In contrast, our findings are more aligned with more recent work that emphasizes anemia as a systemic prognostic factor for mortality rather than a determinant of ISR. Yazji et al. [16] demonstrated that anemic patients with acute coronary syndromes undergoing PCI had significantly higher rates of mortality and stent thrombosis, but ISR was not the main driver of adverse outcomes. Lanser et al. [17] similarly described anemia as a marker of chronic inflammation and comorbidity burden, linking it consistently with poor survival but not uniformly with restenosis. Our study extends this literature by focusing specifically on patients treated exclusively with second-generation DES and by using a clinically driven definition of ISR, showing that, in this cohort and under a clinically driven endpoint definition, we did not detect an independent association between anemia and clinically driven restenosis requiring repeat intervention while anemia remained a robust predictor of long-term mortality.

Several factors may explain the apparent discrepancy between studies linking anemia to ISR and the null association observed here. First, many earlier investigations defined ISR angiographically and often incorporated routine surveillance angiography. Angiographic ISR is more frequent than clinically relevant restenosis and may capture asymptomatic or minimally symptomatic lesions of uncertain prognostic significance. By contrast, we required ischemic symptoms or objective ischemia plus angiographic confirmation, thereby restricting our endpoint to ISR events of clear clinical consequence. This approach likely explains, at least in part, the very low TLR rate observed in our cohort and underscores that anemia does not seem to modulate the risk of clinically driven restenosis after second-generation DES implantation.

Second, differences in stent technology are critical. Associations between anemia and ISR were often reported in the bare-metal stent or first-generation DES eras, when restenosis rates were substantially higher and local inflammatory responses more pronounced. Our cohort consisted exclusively of patients treated with contemporary second-generation DES platforms, in which neointimal proliferation and restenosis rates are markedly reduced [10]. In this setting, restenosis risk appears to be driven predominantly by lesion and procedural characteristics—such as lesion length, vessel size, and stent design—and by systemic inflammatory and metabolic factors rather than hemoglobin levels alone [16, 17, 21]. Our findings support the concept that, once modern DES are used, anemia is no longer a key determinant of target-lesion failure.

Third, methodological rigor and sample size differ across studies. Several prior cohorts included fewer than 1,000 patients and had limited ability to adjust for confounding comorbidities, raising concerns about residual confounding. By contrast, we analyzed more than 4,000 patients from a single high-volume center, applied IPW based on key clinical covariates, and confirmed adequate covariate balance through standardized mean difference plots. Sensitivity analyses using competing-risk models for TLR further demonstrated that the null association between anemia and clinically driven ISR was not explained by the higher mortality in anemic patients. These methodological strengths lend weight to the conclusion that anemia per se is not an independent ISR determinant in the setting of second-generation DES.

While anemia was not linked to ISR, it remained a strong predictor of mortality in our cohort. The adjusted HR of 1.87 for all-cause death is consistent with prior studies and meta-analyses showing anemia as a powerful determinant of adverse outcomes across cardiovascular populations [16, 22–24]. The mechanisms underlying this association are likely multifactorial. Reduced oxygen-carrying capacity increases myocardial vulnerability during ischemic episodes and impairs recovery after infarction [24]. Anemia frequently coexists with chronic kidney disease, diabetes, and systemic inflammation, each independently associated with poor prognosis [12, 17]. Elevated inflammatory cytokines, iron deficiency, and bone marrow dysfunction contribute further to this adverse milieu. In addition, anemic patients often face complex trade-offs in antithrombotic management, with a higher baseline bleeding risk and greater likelihood of treatment modification or discontinuation over time [25]. Our long-term data reinforce the notion that baseline anemia identifies a particularly vulnerable subset of PCI patients whose survival remains compromised despite contemporary stent technology.

A number of hematologic and inflammatory indices beyond hemoglobin—such as red cell distribution width (RDW) and composite blood cell parameters—have been implicated in restenosis risk [18–20, 22]. These markers may better capture chronic inflammation, anisocytosis, or prothrombotic states that influence neointimal proliferation and vascular healing. Our results fit within this framework: anemia as a categorical diagnosis was not associated with clinically driven ISR after second-generation DES implantation, whereas other markers of systemic inflammation and lesion complexity reported in the literature appear to be more relevant for restenosis risk.

The strengths of our study include its large sample size, the exclusive use of second-generation DES, long-term follow-up with a median of five years, and rigorous adjustment for baseline covariates using IPW, supplemented by competing-risk analyses for TLR. Compared with prior smaller cohorts, this study provides one of the most comprehensive evaluations of anemia, clinically driven ISR, and mortality in an all-comers DES-era population from a high-volume tertiary center. The use of a clinically driven ISR endpoint enhances the clinical applicability of our findings by focusing on events with clear symptomatic or ischemic significance.

Our findings should be interpreted in the context of several important limitations. First, the retrospective, single-center design precludes causal inference and limits generalizability to other populations and healthcare settings. The exclusion of patients with prior PCI or CABG, while intended to create a de-novo lesion cohort, further restricts the applicability of our findings to the common real-world population with prior revascularization.

Second, and most critically, our primary endpoint of clinically driven TLR, while pragmatic, is subject to ascertainment bias. The absence of routine angiographic follow-up means that asymptomatic or minimally symptomatic restenosis was not captured. This, combined with the low overall number of TLR events (*n* = 73), resulted in limited statistical power for our primary analysis. The near-significant p-value and wide confidence interval for the adjusted HR suggest that we cannot exclude a modest association between anemia and TLR, and our study should be considered underpowered for this endpoint.

Third, despite the use of IPW to balance a wide range of covariates, residual confounding from unmeasured variables remains possible. We were unable to adjust for several factors that could influence both anemia and outcomes, including the specific etiology and severity of anemia, changes in hemoglobin over time, frailty, nutritional status, and details of antiplatelet therapy (type, duration, and adherence); therefore, we could not assess whether anemia-associated modification of dual antiplatelet therapy influenced restenosis-related revascularization or mortality.

Fourth, our definition of anemia as a binary variable based on a single baseline measurement is a simplification. It does not account for anemia severity (e.g., mild vs. moderate/severe), its underlying cause (e.g., iron deficiency vs. anemia of chronic disease), or its dynamic nature over the five-year follow-up. Different anemia phenotypes may have distinct impacts on vascular biology and mortality, and this heterogeneity could have diluted or obscured true associations with TLR. We lacked granular data on clinical presentation subtypes (NSTEMI, unstable angina, stable angina) and therefore could not adjust for potential differences in ischemic risk between these groups.

Finally, as a single-center study from Iran, the characteristics of our cohort, including a high prevalence of diabetes and opium use, may differ from those in other regions, potentially influencing the generalizability of our findings.

The clinical implications of these findings are twofold. First, baseline anemia in patients undergoing PCI with second-generation DES should be viewed primarily as a systemic prognostic marker rather than as a driver of local restenosis. Anemic patients merit intensified risk stratification, thorough evaluation for reversible causes (including iron deficiency, occult gastrointestinal blood loss, renal dysfunction, and chronic inflammatory conditions), and careful optimization of secondary prevention strategies. Whether targeted correction of anemia improves long-term survival in this setting remains an important question for future randomized trials. Second, in this cohort using symptom/ischemia-guided follow-up and a clinically driven TLR endpoint, we did not observe a signal to support baseline anemia alone as an indication for intensified ISR surveillance beyond standard symptom/ischemia-guided care; attention should instead remain focused on established restenosis determinants such as lesion complexity and vessel size.

Future research should aim to integrate anemia into comprehensive prognostic models alongside renal dysfunction, diabetes, and inflammatory markers in PCI populations treated with modern DES. Large-scale prospective registries and randomized studies of anemia correction strategies will be crucial to clarify whether anemia is simply a marker of risk or a modifiable treatment target. In addition, studies stratifying outcomes by anemia etiology and severity may yield more precise insights into which subsets of anemic patients derive the greatest prognostic benefit from targeted management.

## Conclusions

In conclusion, this large cohort study demonstrates that while baseline anemia is a powerful and independent predictor of long-term mortality after PCI with second-generation DES, it was not associated with an increased risk of clinically driven TLR. For clinicians, this distinction is important: anemia robustly identifies a high-risk patient warranting aggressive systemic risk factor management and investigation into its underlying causes. However, given the limitations of our primary endpoint and the low event rate, our findings should not be interpreted as definitive evidence that anemia plays no role in restenosis pathophysiology. Future studies with prospective design, systematic assessment of ischemia, and serial measurement of hematinic and inflammatory markers are needed to definitively clarify the relationship between anemia, its various phenotypes, and long-term coronary stent failure.

## Supplementary Information

Below is the link to the electronic supplementary material.

Supplementary Material 1.

## Data Availability

The datasets generated and/or analysed during the current study are available from the corresponding author on reasonable request, subject to institutional approvals and a data access agreement.

## Abbreviations

CAD: Coronary artery disease
PCI: Percutaneous coronary intervention
DES: Drug-eluting stent
ISR: In-stent restenosis
TLR: Target lesion revascularization
TVR: Target vessel revascularization
MACCE: Major adverse cardiac and cerebrovascular events
LVEF: Left ventricular ejection fraction
IPW: Inverse probability weighting
CKD: Chronic kidney disease

## Declarations

### Use of generative artificial intelligence

Generative artificial intelligence (ChatGPT, OpenAI) was used only to assist in language editing of the manuscript. The authors verified all content for accuracy, scientific validity, and originality, and take full responsibility for the final submitted version. No AI tools were used for data analysis, statistical modelling, or drawing scientific conclusions.
