# Supplementary material for: Anemia and long-term outcomes after second-generation drug-eluting stent implantation: a retrospective cohort study of mortality and clinical restenosis
Source: Egypt Heart J. 2026 Apr 28;78:28. doi: 10.1186/s43044-026-00725-8 (PMC13125393; doi:10.1186/s43044-026-00725-8)
Supplement: Supplementary file 1 — Supplementary Material 1. [file 43044_2026_725_MOESM1_ESM.docx]

**Supplementary Table 1:** Fine-Gray Competing Risk Analysis for Target Lesion Revascularization (TLR) with Death as Competing Event

| \| Variable \| Subdistribution Hazard Ratio (SHR) \| 95% Confidence Interval \| p-value \| \| --- \| --- \| --- \| --- \| \| **Anemia** (yes vs. no) \| 0.38 \| 0.11–1.31 \| 0.124 \| \| **Age** (per year increase) \| 1.01 \| 0.98–1.04 \| 0.352 \| \| **Gender** (female vs. male) \| 0.94 \| 0.55–1.61 \| 0.821 \| \| **Body Mass Index** (per unit increase) \| 1.00 \| 0.95–1.05 \| 0.912 \| \| **eGFR** (per 10 mL/min/1.73m² decrease) \| 1.12 \| 0.94–1.33 \| 0.198 \| \| **Diabetes** (yes vs. no) \| 0.86 \| 0.52–1.42 \| 0.554 \| \|  \|  \|  \|  \| \| **Hypertension** (yes vs. no) \| 1.28 \| 0.79–2.07 \| 0.318 \| \| **Dyslipidemia** (yes vs. no) \| 0.96 \| 0.59–1.56 \| 0.868 \| \| **Current Smoking** (yes vs. no) \| 0.91 \| 0.55–1.51 \| 0.714 \| \| **Family History of CAD** (yes vs. no) \| 0.67 \| 0.33–1.36 \| 0.267 \| \| **Previous Myocardial Infarction** (yes vs. no) \| 2.58 \| 1.45–4.59 \| 0.001 \| \| **History of CVA/TIA** (yes vs. no) \| 1.48 \| 0.45–4.87 \| 0.516 \| \| **History of Renal Failure** (yes vs. no) \| 1.68 \| 0.38–7.42 \| 0.495 \| \| **History of Chronic Lung Disease** (yes vs. no) \| 0.92 \| 0.13–6.52 \| 0.932 \| \| **History of CHF** (yes vs. no) \| 0.83 \| 0.12–5.74 \| 0.851 \| \| **LAD Territory** (yes vs. no) \| 1.05 \| 0.65–1.70 \| 0.842 \| \| **ACC/AHA Lesion Class** (B2/C vs. A/B1) \| 1.55 \| 0.49–4.90 \| 0.455 \| \| **Lesion Length** (per mm increase) \| 1.01 \| 0.99–1.03 \| 0.312 \| \| **Thrombus Suction** (yes vs. no) \| 1.15 \| 0.50–2.64 \| 0.742 \| \| **Bifurcation Lesion** (yes vs. no) \| 1.02 \| 0.54–1.93 \| 0.952 \| \| **Overlap Stent** (yes vs. no) \| 1.55 \| 0.73–3.29 \| 0.253 \| \| **Post-dilation** (yes vs. no) \| 0.93 \| 0.57–1.52 \| 0.772 \| \| **Stent Diameter** (per mm increase) \| 0.84 \| 0.52–1.36 \| 0.478 \| \| **First Stent Length** (per mm increase) \| 1.01 \| 0.99–1.03 \| 0.384 \| \| **Number of Stents** (> 1 vs. 1) \| 1.42 \| 0.44–4.58 \| 0.558 \| \| **Simultaneous PCI** (yes vs. no) \| 1.26 \| 0.71–2.24 \| 0.432 \| |
| --- | --- | --- | --- | --- | --- | --- | --- | --- | --- | --- | --- | --- | --- | --- | --- | --- | --- | --- | --- | --- | --- | --- | --- | --- | --- | --- | --- | --- | --- | --- | --- | --- | --- | --- | --- | --- | --- | --- | --- | --- | --- | --- | --- | --- | --- | --- | --- | --- | --- | --- | --- | --- | --- | --- | --- | --- | --- | --- | --- | --- | --- | --- | --- | --- | --- | --- | --- | --- | --- | --- | --- | --- | --- | --- | --- | --- | --- | --- | --- | --- | --- | --- | --- | --- | --- | --- | --- | --- | --- | --- | --- | --- | --- | --- | --- | --- | --- | --- | --- | --- | --- | --- | --- | --- | --- | --- | --- | --- | --- | --- | --- | --- |
